# MYD88/TRIF Signaling, Pluripotency and Klotho Regulation in the Intestine, Kidneys, Liver, and Lungs of a Septic Mouse Model

**DOI:** 10.3390/cimb48070660

**Published:** 2026-06-26

**Authors:** Maria Erodotou, Alkistis Kapelouzou, Konstantinos S. Mylonas, Ioanna Soukouli, John N. Boletis, Gerasimos Tsourouflis, Theodore Liakakos, Dimitrios Schizas

**Affiliations:** 1 First Department of Surgery, National and Kapodistrian University of Athens, Laikon General Hospital, 106 79 Athens, Greece; konstantinos.mylonas@emory.edu (K.S.M.); theodlia@gmail.com (T.L.); schizasad@gmail.com (D.S.); 2 Clinical, Experimental Surgery and Translational Research, Biomedical Research Foundation Academy of Athens, 106 79 Athens, Greece; akapelouzou@gmail.com; 3 Department of Nephrology and Renal Transplantation, National and Kapodistrian University of Athens, Laikon General Hospital, 106 79 Athens, Greece; ioanna.s.soukouli@gmail.com (I.S.); inboletis@gmail.com (J.N.B.); 4 Second Department of Propaedeutic Surgery, National and Kapodistrian University of Athens, Laikon General Hospital, 106 79 Athens, Greece; gerasimos.ts@gmail.com

**Keywords:** sepsis, CLP model, TLR’s, MYD88 pathway, TRIF pathway, intestine, kidney, liver, lung

## Abstract

Sepsis is a life-threatening condition characterized by a dysregulated host response to infection, leading to multi-organ dysfunction. Toll-like receptor signaling via MYD88- and TRIF-dependent pathways plays a central role in this process; however, its temporal and tissue-specific dynamics remain incompletely understood. The aim of this study was to investigate time-dependent transcriptional changes in MYD88- and TRIF-dependent signaling pathways across multiple organs in a murine model of sepsis. mRNA expression of MYD88, IRAK1, IRAK4, NF-kB, CCL4, CCL20, CCR2, IFN-β, IFN-γ, TNF-α, IL-1β, IL-2, IL-4, IL-8, IL-10, IL-18, Klotho, KLF4, HOXA5, NANOG and HIF1α was quantified using qRT-PCR in intestinal, kidney, liver and lung tissues at 24, 48, and 72 h following cecal ligation and puncture-induced sepsis in male C57BL/6J mice. Significant upregulation of innate immune signaling molecules, cytokines, chemokines, and interferon-related genes was observed in all tissues compared with controls. Genes associated with hypoxia and cellular regulation were also increased. These responses were tissue-specific and progressively intensified over time. Sepsis represents a dynamic, time-dependent, and tissue-specific process characterized by sustained activation of immune and hypoxic pathways, providing potential targets for time-stratified therapeutic strategies.

## 1. Introduction

Sepsis is a potentially life-threatening condition characterized by a dysregulated host response to infection that results to multi-organ dysfunction. Toll-like receptor (TLR) activation represents a central mechanism of the innate immune response during sepsis, primarily through the engagement of the MYD88- (Myeloid Differentiation Primary Response 88) and TRIF (TIR domain containing adaptor inducing interferon)-dependent signaling pathways [1]. TLR stimulation produces adaptor proteins such as MYD88 to recruit downstream kinases, including IRAK1 (Interleukin-1 Receptor–Associated Kinase 1) and IRAK4 (Interleukin-1 Receptor–Associated Kinase 4), leading to the activation of NF-κB (Nuclear factor kappa-light-chain-enhancer of activated B cells), which controls the transcription of a broad spectrum of inflammatory mediators [2]. This signaling cascade promotes the expression of chemokines such as CCL4 (Chemokine [C-C motif] ligand 4), CCL20 (Chemokine [C-C motif] ligand 20) and CCR2 (C-C chemokine receptor type 2), which are necessary for leukocyte recruitment and immune cell trafficking, and the expression of pro- and anti-inflammatory cytokines including TNF-α (Tumor Necrosis Factor alpha), IL-1β (Interleukin 1β), IL-2 (Interleukin 2), IL-4 (Interleukin 4), IL-8 (Interleukin 8), IL-10 (Interleukin 10), and IL-18 (Interleukin 18) [3,4].

Similarly, the TRIF-dependent signaling pathway, particularly downstream of TLR3 and TLR4 activation, contributes to the induction of interferons type I and II, including IFN-β (Interferon beta) and IFN-γ (Interferon gamma) [3,4]. Interferons, particularly type I interferons, play a central role in antiviral immunity and responses to intracellular pathogens, while also contributing to immune modulation during sepsis [3,4]. Outside the scope of traditional inflammatory mediators, evidence shows that sepsis-associated TLR signaling also influences the transcription of genes involved in cellular stress responses, endothelial integrity, hypoxia adaptation and tissue repair, such as Klotho, KLF4 (Krüppel-like factor 4), HOXA5 (Homeobox A5), NANOG, and HIF-1a (Hypoxia-Inducible Factor 1 alpha) [4]. Overall, the transcriptional regulation of these immune and stress-related genes offers pivotal insight into the complex, organ-specific responses observed during sepsis.

To investigate these mechanisms in a clinically relevant context, animal models are necessary. The Cecal Ligation and Puncture (CLP) model is widely used as the gold standard for studying polymicrobial sepsis due to its ability to mimic the human pathophysiology of sepsis [5,6].

The aim of this study is to quantify the mRNA expression of a panel of genes, including MYD88, IRAK1, IRAK4, NF-kB, CCL4, CCL20, CCR2, IFN-β, IFN-γ, TNF-α, IL-1β, IL-2, IL-4, IL-8, IL-10, IL-18, Klotho, KLF4, HOXA5, NANOG and HIF1α, relative to GAPDH in intestinal, kidney, liver and lung tissues from the early and late phases of CLP-induced sepsis in mice. The selected genes were categorized based on their functional roles, including (i) signaling molecules (MYD88, IRAK1, IRAK4, NF-κB), (ii) chemokines and receptors (CCL4, CCL20, CCR2), (iii) cytokines and interferons (TNF-α, IL-1β, IL-2, IL-4, IL-8, IL-10, IL-18, IFN-β, IFN-γ), and (iv) genes involved in hypoxia, cellular stress, and tissue repair (Klotho, KLF4, HOXA5, NANOG, HIF-1α). These findings aim to clarify the rate of increase in MYD88 and TRIF pathways during sepsis. A deeper understanding of these results may reveal potential therapeutic targets for modulating the immune response during sepsis, with the goal of improving clinical outcomes and reducing multi-organ dysfunction.

## 2. Materials and Methods

### 2.1. Animal Model and Experimental Procedure

The present study was based on data obtained from previously conducted experiments by Bakopoulos et al. at the Biomedical Research Foundation Academy of Athens (BRFAA), in accordance with approved experimental protocols (protocol no. 2838/21-07-2011) [7]. Sepsis was induced in 72 male C57BL/6J mice, aged 12–14 weeks and weighing 20–25 g, using the CLP method [7]. Animals were divided into two groups: septic (S), subjected to cecal ligation and puncture (CLP), and sham-operated controls (SH), which underwent identical surgical procedures without ligation or puncture [7]. The animal model, experimental procedures, and CLP-induced sepsis protocol employed in these studies have been previously described in detail by our research group and are based on already published and ethically approved experiment [7]. Briefly, these studies utilized a well-established murine CLP model of sepsis, with standardized housing conditions, perioperative care, and predefined endpoints [7]. Mice from both septic and sham groups were sacrificed at 24, 48, and 72 h post-operation [7]. The time points (24, 48, and 72 h) were selected to capture early, intermediate, and late phases of sepsis progression in the CLP model, which are associated with escalating inflammatory responses and increased mortality. The data generated from these experiments were reanalyzed and integrated into the present work to further investigate time-dependent inflammatory and gene expression responses under septic conditions.

Successful induction of sepsis in the CLP model has been previously validated in the original experimental studies [7], which demonstrated consistent clinical deterioration, inflammatory activation, and organ-specific responses characteristic of polymicrobial sepsis.

### 2.2. Real-Time Polymerase Chain Reaction (PCR)

Total RNA was extracted using Trizol reagent according to the manufacturer’s protocol (Life Technologies-Invitrogen, Carlsbad, CA, USA). A total of 1 μg of total RNA was used to perform reverse transcription and synthesis of circular DNA (cDNA) using MMLV reverse transcriptase. RNA quality was assessed based on purity and amplification consistency. Although RNA integrity number (RIN) values were not formally recorded, all samples demonstrated consistent Ct values and amplification efficiency, supporting adequate RNA quality for qRT-PCR analysis. The primers for MYD88, IRAK1, IRAK4, NF-kB, CCL4, CCL20, CCR2, IFN-β, IFN-γ, TNF-α, IL-1β, IL-2, IL-4, IL-8, IL-10, IL-18, Klotho, KLF4, HOXA5, NANOG and HIF1α used for PCR were designed and produced by Integrated DNA Technologies (Leuven, Belgium). Supplementary Table S1.

QRT-PCR was performed using the LightCycler 480 (Roche, Mannheim, Germany). Briefly, each reaction of 20 μL total volume contained 2 μL cDNA (20 ng total RNA), each primer (gene detection) 200 nM and 10 μL of Kapa Sybr Fast qPCR mix (KAPA BIO, Boston, MA, USA). After an initial denaturation step at 95 °C for 10 min, the PCR conditions were: 95 °C × 30 s, 60 °C × 40 s, 72 °C × 40 s, 40 cycles. All samples were repeated in duplicate and the mean Ct value for each sample was used for data analysis. Relative gene expression levels were determined using the 2^−^ΔΔCt method, normalized to GAPDH, and expressed as a percentage relative to control samples, as previously described [8].

### 2.3. Statistical Analysis

The statistical processing of the experimental data was carried out with the GraphPad Prism software, version 4.03 (GraphPad Software, San Diego, CA, USA). The comparison of the groups was made through one-way analysis of variance (one-way ANOVA). In cases where a statistically significant difference was found, a *t*-test was followed to identify the individual differences between specific groups. The results are presented as mean ± standard deviation (mean ± SD). Statistical significance is determined at a 95% confidence level and is considered significant when the *p*-value is less than 0.05 (*p* < 0.05).

## 3. Results

**Figure 1:**
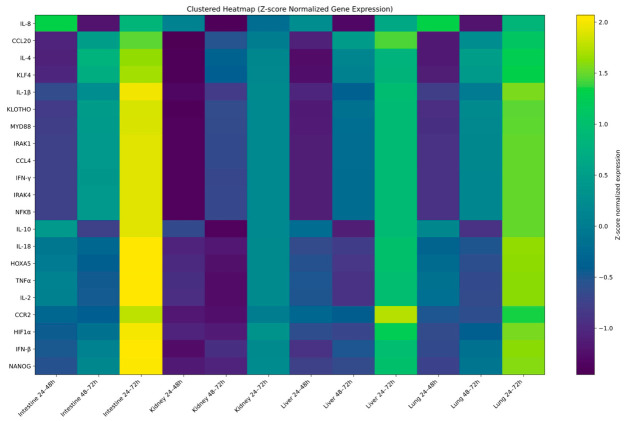
**Clustered heatmap of time-dependent gene expression changes across tissues in septic mice.** Gene expression values were Z-score normalized and hierarchically clustered to highlight patterns across tissues and time intervals (24–48 h, 48–72 h, and 24–72 h). Rows represent genes and columns represent tissue/time combinations. The heatmap reveals coordinated, tissue-specific transcriptional responses and progressive activation of inflammatory and hypoxia-related pathways during sepsis.

The mRNA expression levels of MYD88- and TRIF-related signaling molecules, cytokines, chemokines, and stem cell-associated genes at 24, 48 and 72 h in all tested tissues (intestinal, kidneys, liver and lungs) in sham (SH) and septic (S) groups are presented in Supplementary Tables S2–S5. QRT-PCR of the tested genes showed a significant increase in the mRNA expression of MYD88, IRAK1, IRAK4 and NF-κB, increased expression of the chemokines CCL4, CCL20 and CCR2 receptor, significant induction of the cytokines TNF-α, IL-1β, IL-2, IL4, IL-8, IL10 and IL-18, increased expression of IFN-β and IFN-γ and significant induction of Klotho, as well as KLF4, HOXA5, NANOG and HIF-1α genes in the septic models from 24 h to 72 h compared to the control groups. To enhance visualization of global transcriptional patterns, gene expression data were Z-score normalized and subjected to hierarchical clustering prior to heatmap generation (Figure 1).

### 3.1. Intestine Tissue

**Figure 2:**
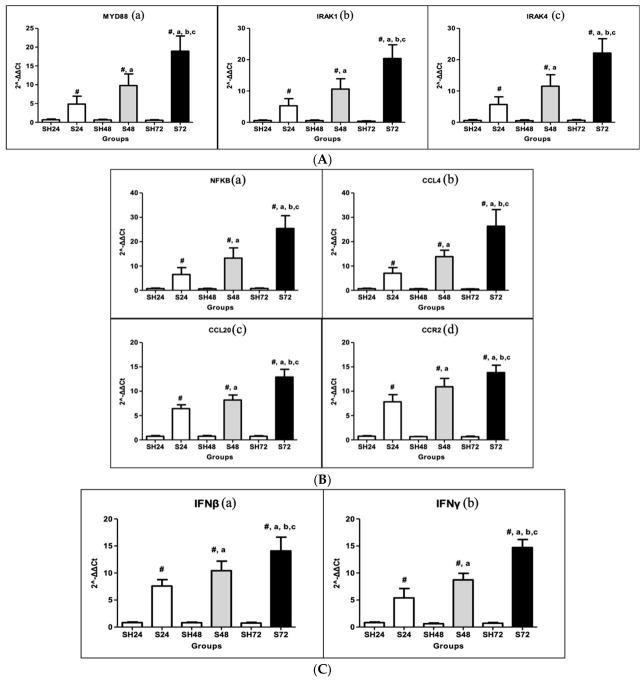

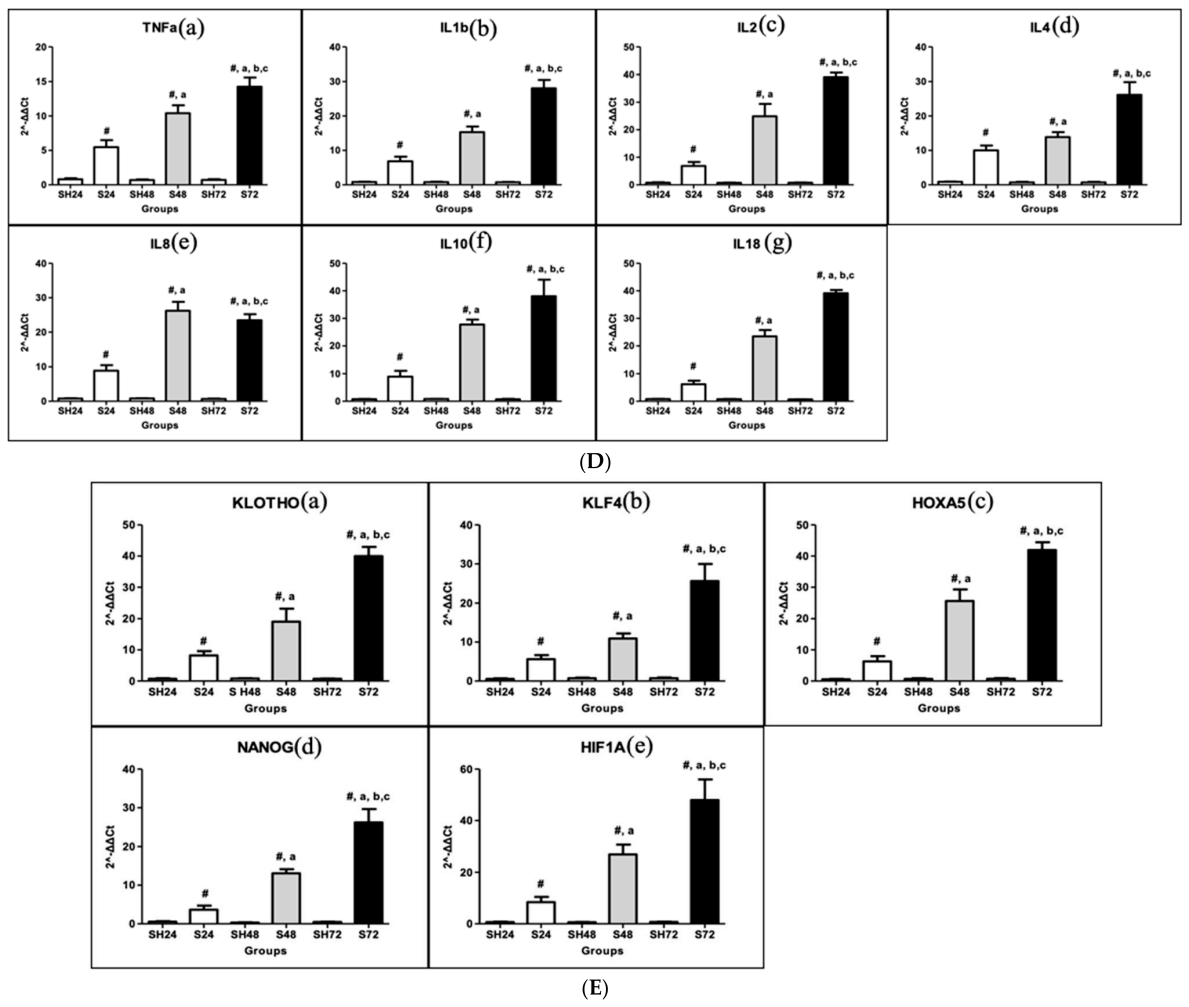
(**A**–**E**). Relative mRNA expression levels in intestinal tissue of MYD88, IRAK1, IRAK4, NF-κB, CCL4, CCL20, CCR2, IFN-β, IFN-γ, TNF-α, IL-1β, IL-2, IL-4, IL-8, IL-10, IL-18, Klotho, KLF4, HOXA5, NANOG and HIF1α in sham (SH) and septic (S) groups at 24, 48, and 72 h. Data are presented as mean ± standard deviation (SD), with *n* = 12 mice per group. Statistical analysis was performed using one-way ANOVA with *p* < 0.05 considered statistically significant. Statistical annotations are defined as follows: (#) indicates significant differences between sham (SH) and septic (S) groups at the corresponding time points (24, 48, and 72 h); (a) indicates a statistically significant difference between the septic group at 24 h (S24) and septic group at 48 h (S48); (b) indicates a statistically significant difference between the septic group at 24 h (S24) and septic group at 72 h (S72); and (c) indicates a statistically significant difference between the septic group at 48 h (S48) and septic group at 72 h (S72).

As shown in Figure 2A–E and Table 1, MYD88, IRAK1, and IRAK4 mRNA expression levels in septic groups exhibited a consistent and significant increase over time, with marked elevations observed from 24 to 48 h, from 48 to 72 h, and cumulatively from 24 to 72 h. Similarly, within the septic models, NFκB, CCL4, and CCL20 gene expression levels increased significantly across all examined time intervals, indicating sustained activation throughout the septic course. In contrast, CCR2 expression was significantly elevated between 24 and 48 h of sepsis. Although CCR2 levels remained increased from 48 to 72 h, the magnitude of this rise was less pronounced. Overall, CCR2 expression demonstrated a robust and sustained upregulation over the entire 24–72 h septic period. IFN-β and IFN-γ expression levels increased significantly among the septic models from 24 to 48 h, with a similar upward trend observed from 48 to 72 h and a more pronounced increase across the full 24 to 72 h interval. TNFα, IL-2, IL-8, IL-10, and IL-18 mRNA levels showed a marked amplification during the early phase (24–48 h) of sepsis, followed by a modest yet statistically significant elevation from 48 to 72 h, resulting in a pronounced cumulative increase over the 24–72 h period. In contrast, IL-1β and IL-4 exhibited a progressive and continuous upregulation across all time intervals. Furthermore, Klotho, KLF4, NANOG and HIF1α mRNA expression levels in septic models showed significant increases during all time intervals. Conversely, HOXA5 expression demonstrated a significant increase from 24 to 48 h, a less pronounced but still significant elevation from 48 to 72 h, and a more substantial overall increase when comparing 24 to 72 h.

### 3.2. Kidney Tissue

**Figure 3:**
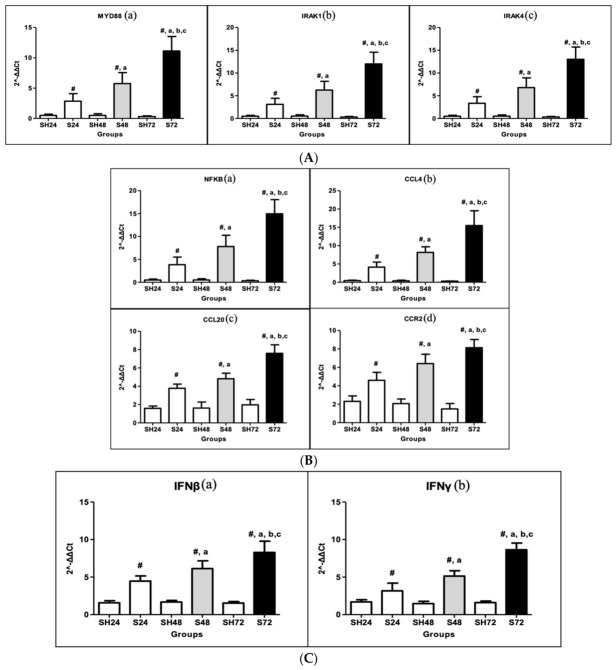

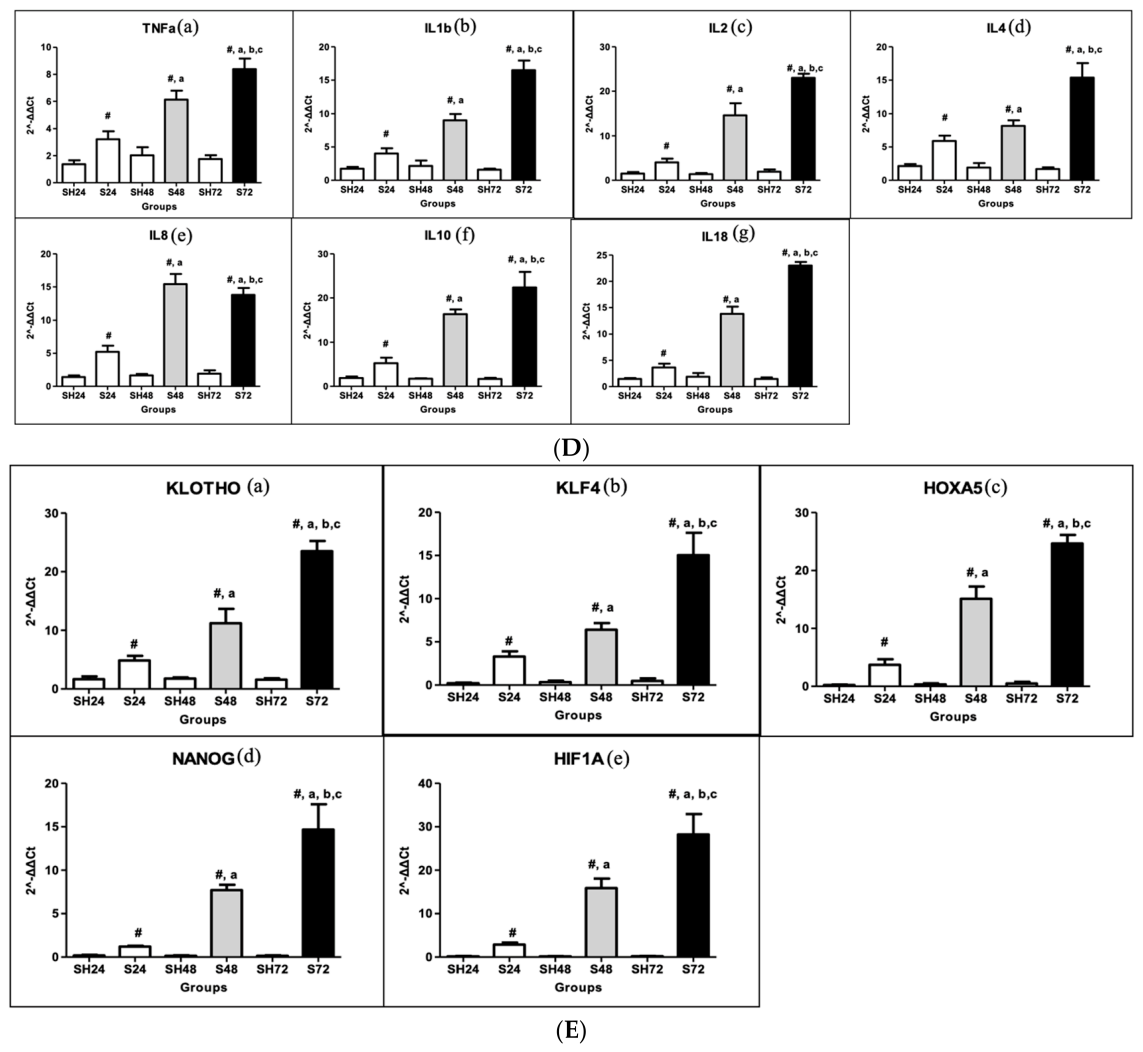
(**A**–**E**). Relative mRNA expression levels in kidney tissue of MYD88, IRAK1, IRAK4, NF-κB, CCL4, CCL20, CCR2, IFN-β, IFN-γ, TNF-α, IL-1β, IL-2, IL-4, IL-8, IL-10, IL-18, Klotho, KLF4 HOXA5, NANOG and HIF1α in sham (SH) and septic (S) groups at 24, 48, and 72 h. Data are presented as mean ± standard deviation (SD), with *n* = 12 mice per group. Statistical analysis was performed using one-way ANOVA with *p* < 0.05 considered statistically significant. Statistical annotations are defined as follows: (#) indicates significant differences between sham (SH) and septic (S) groups at the corresponding time points (24, 48, and 72 h); (a) indicates a statistically significant difference between the septic group at 24 h (S24) and septic group at 48 h (S48); (b) indicates a statistically significant difference between the septic group at 24 h (S24) and septic group at 72 h (S72); and (c) indicates a statistically significant difference between the septic group at 48 h (S48) and septic group at 72 h (S72).

Insights from Figure 3A–E and Table 2 demonstrate that MYD88, IRAK1, and IRAK4 gene expression levels were significantly upregulated in septic groups across all evaluated time intervals, including 24–48 h, 48–72 h and 24–72 h. Similarly, NFκB, CCL4, and CCL20 mRNA expressions in septic mice increased significantly from 24 to 48 h, with this upward trend persisting from 48 to 72 h and resulting in a pronounced elevation over the entire 24–72 h period. In contrast, CCR2 expression in septic mice exhibited a modest but significant increase during the 24–48 h interval. From 48 to 72 h, CCR2 levels remained elevated but showed a less pronounced rise, whereas the cumulative comparison from 24 to 72 h revealed a more substantial overall increase. Regarding interferon-related genes, IFN-β and IFN-γ expression levels in septic models were significantly enhanced from 24 to 48 h, with similar increases observed from 48 to 72 h and across the full 24–72 h time frame. Additionally, TNFα, IL-2, IL-8, IL-10, and IL-18 mRNA levels in septic groups demonstrated marked upregulation during the early phase (24–48 h), followed by a less extensive yet statistically significant elevation from 48 to 72 h, culminating in a significant overall increase from 24 to 72 h. In contrast, IL-1β and IL-4 showed a consistent and significant increase across all time intervals. Finally, Klotho, KLF4 and NANOG mRNA expression levels in septic mice increased substantially from 24 to 48 h, with this upward trend continuing from 48 to 72 h and remaining significant across the entire 24–72 h period. Conversely, HOXA5 and HIF1α expression levels rose significantly from 24 to 48 h; although their increase from 48 to 72 h was less pronounced, it remained statistically significant. Overall, HOXA5 and HIF1α expressions demonstrated a notable cumulative upregulation when comparing 24 to 72 h.

### 3.3. Liver Tissue

**Figure 4:**
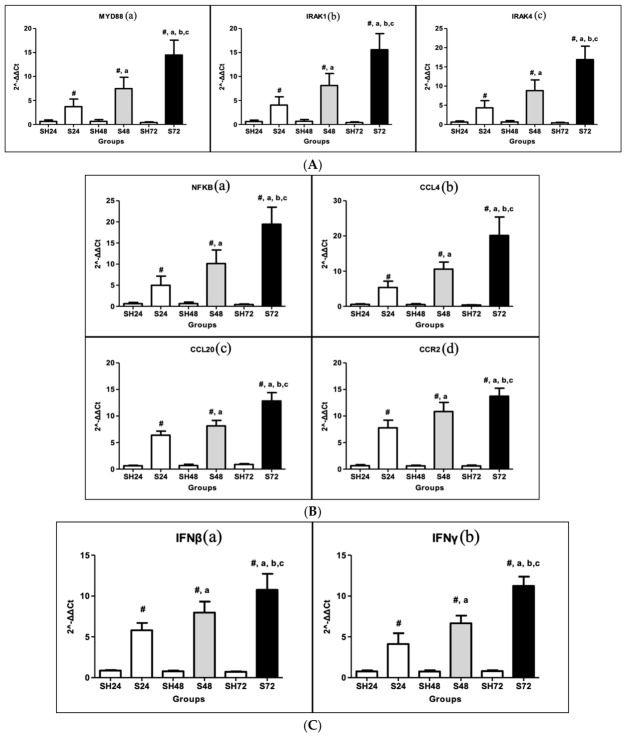

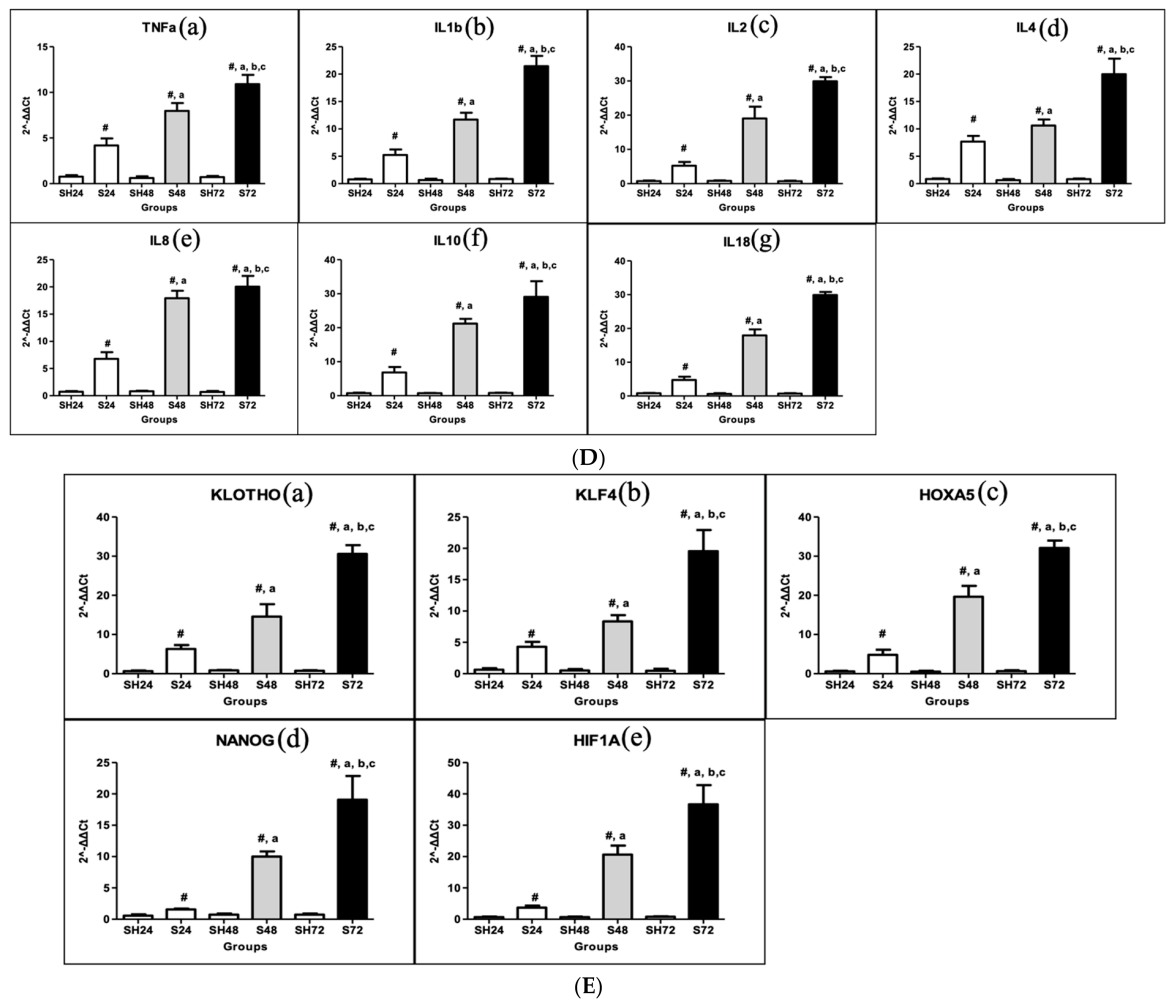
(**A**–**E**). Relative mRNA expression levels in liver tissue of MYD88, IRAK1, IRAK4, NF-κB, CCL4, CCL20, CCR2, IFN-β, IFN-γ, TNF-α, IL-1β, IL-2, IL-4, IL-8, IL-10, IL-18, Klotho, KLF4, HOXA5, NANOG and HIF1α in sham (SH) and septic (S) groups at 24, 48, and 72 h. Data are presented as mean ± standard deviation (SD), with *n* = 12 mice per group. Statistical analysis was performed using one-way ANOVA with *p* < 0.05 considered statistically significant. Statistical annotations are defined as follows: (#) indicates significant differences between sham (SH) and septic (S) groups at the corresponding time points (24, 48, and 72 h); (a) indicates a statistically significant difference between the septic group at 24 h (S24) and septic group at 48 h (S48); (b) indicates a statistically significant difference between the septic group at 24 h (S24) and septic group at 72 h (S72); and (c) indicates a statistically significant difference between the septic group at 48 h (S48) and septic group at 72 h (S72).

Data from Figure 4A–E and Table 3 indicate that MYD88, IRAK1, and IRAK4 expression levels in septic groups were significantly increased across all evaluated time intervals, including 24–48 h, 48–72 h, and cumulatively from 24 to 72 h. Similarly, among septic mice, NFκB, CCL4, and CCL20 expression levels rose markedly from 24 to 48 h, with this upward trend continuing from 48 to 72 h and resulting in a pronounced elevation over the entire 24–72 h period. In contrast, CCR2 expression increased significantly during the 24–48 h interval. Although CCR2 levels continued to rise from 48 to 72 h, the magnitude of this increase was more modest, while the cumulative comparison from 24 to 72 h revealed a more pronounced overall elevation. IFN-β and IFN-γ mRNA expressions in septic groups increased significantly from 24 to 48 h, with similar enhancements observed across all time periods. Additionally, TNFα, IL-2, IL-8, IL-10, and IL-18 expression levels showed a prominent increase during the early phase of sepsis (24–48 h), followed by a less dramatic yet statistically significant elevation from 48 to 72 h, culminating in a substantial overall increase from 24 to 72 h. IL-1β and IL-4 showed a consistent and significant increase across all time periods of sepsis. Finally, Klotho, KLF4, and NANOG mRNA expression levels in septic mice demonstrated marked upregulation across all time intervals. Conversely, HOXA5 and HIF1α expression levels increased significantly from 24 to 48 h; although their rise from 48 to 72 h was more modest, it remained statistically significant. Overall, hOXA5 and hIF1α exhibited a pronounced cumulative increase when comparing 24 to 72 h.

### 3.4. Lung Tissue

**Figure 5:**
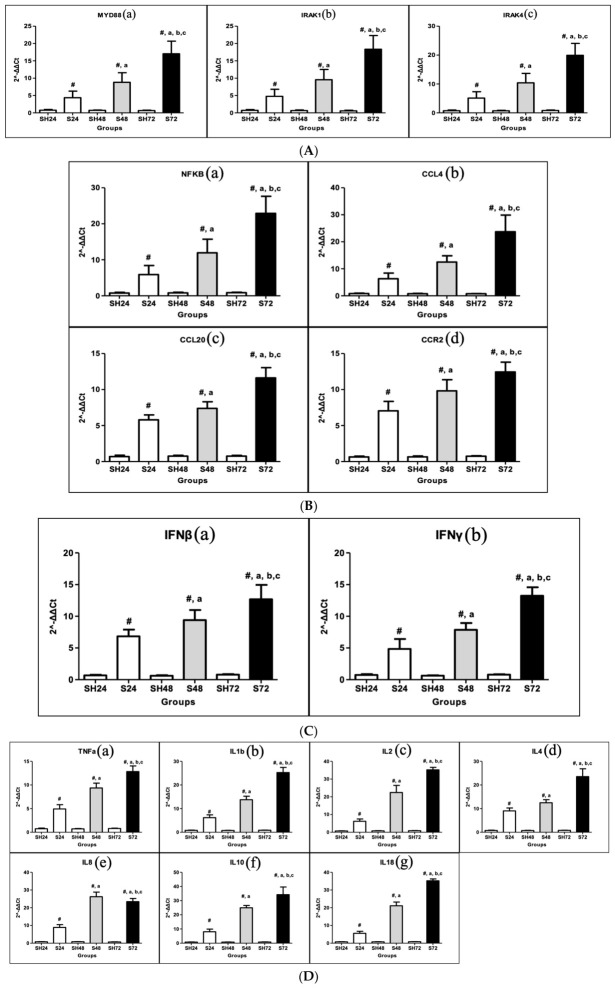

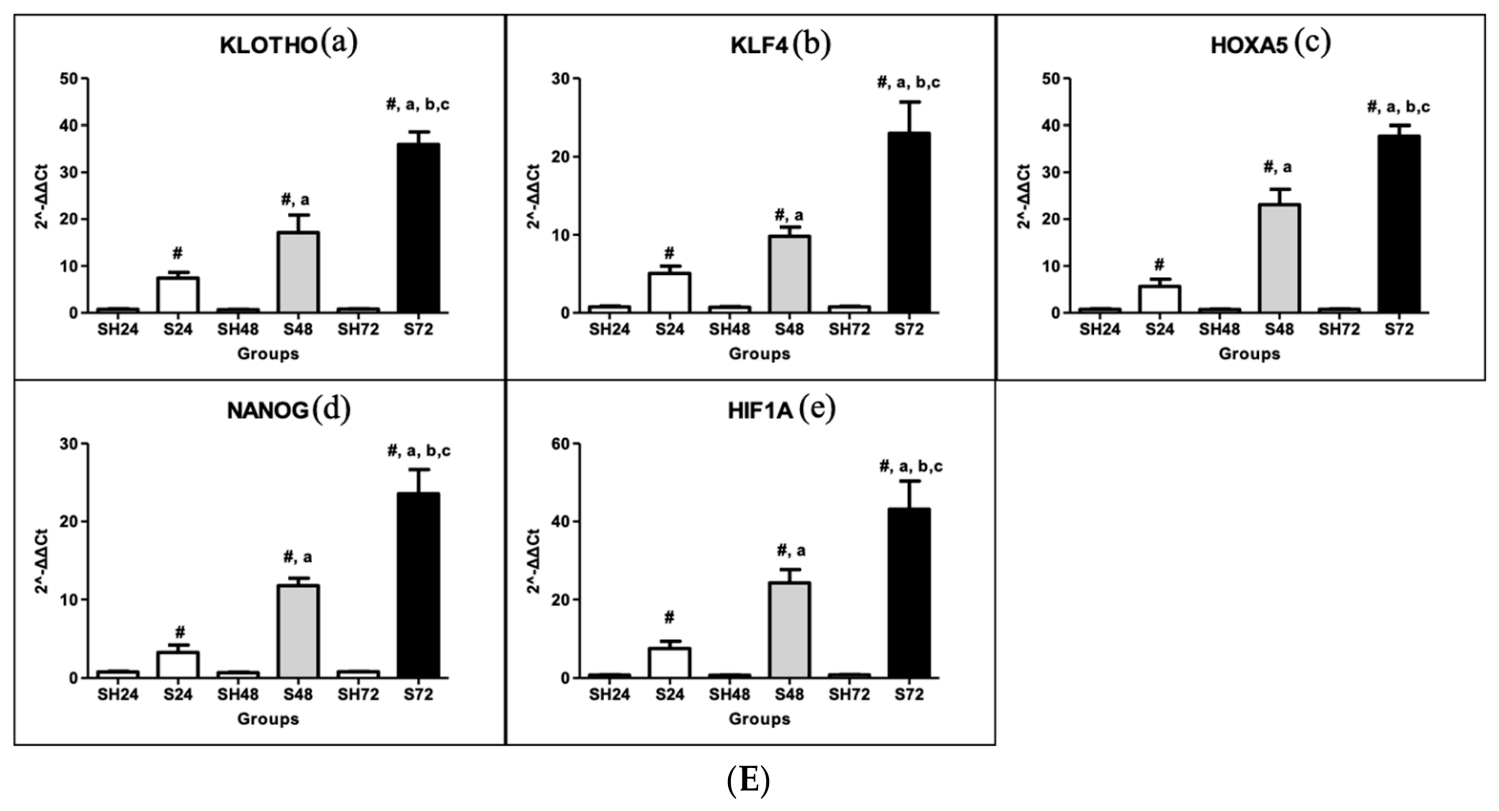
(**A**–**E**). Relative mRNA expression levels in lung tissue of MYD88, IRAK1, IRAK4, NF-κB, CCL4, CCL20, CCR2, IFN-β, IFN-γ, TNF-α, IL-1β, IL-2, IL-4, IL-8, IL-10, IL-18, Klotho, KLF4, HOXA5, NANOG and HIF1α in sham (SH) and septic (S) groups at 24, 48, and 72 h. Data are presented as mean ± standard deviation (SD), with *n* = 12 mice per group. Statistical analysis was performed using one-way ANOVA with *p* < 0.05 considered statistically significant. Statistical annotations are defined as follows: (#) indicates significant differences between sham (SH) and septic (S) groups at the corresponding time points (24, 48, and 72 h); (a) indicates a statistically significant difference between the septic group at 24 h (S24) and septic group at 48 h (S48); (b) indicates a statistically significant difference between the septic group at 24 h (S24) and septic group at 72 h (S72); and (c) indicates a statistically significant difference between the septic group at 48 h (S48) and septic group at 72 h (S72).

Outcomes from Figure 5A–E and Table 4 show that MYD88, IRAK1, and IRAK4 expression levels in septic models were significantly enhanced across all time periods. Likewise, among septic mice, NFκB, CCL4, and CCL20 levels rose notably from 24 to 48 h and this upward trend continued from 48 to 72 h, resulting in a pronounced augmentation over the 24–72 h period. During 24 to 48 h of sepsis, CCR2 expression increased significantly. Although CCR2 levels continued to rise from 48 to 72 h, the magnitude of this increase was less, while the overall comparison from 24 to 72 h showed more prominent elevation. From 24 to 48 h, IFN-β and IFN-γ levels in septic groups increased significantly, with similar enhancements observed across all time intervals. Moreover, TNFα, IL-2, IL-8, IL-10, and IL-18 expression levels revealed a noteworthy increase during the early phase (24–48 h), followed by a less pronounced yet statistically significant elevation from 48 to 72 h, culminating in a significant overall increase from 24 to 72 h. Lastly, Klotho, KLF4, NANOG, and HIF1α mRNA expression levels in septic mice showed significant increases across all time periods, highlighting their sustained activation during sepsis progression. Conversely, HOXA5 expression demonstrated a significant increase from 24 to 48 h, a modest but still significant elevation from 48 to 72 h, and a more substantial overall increase during 24 to 72 h.

## 4. Discussion

The present study builds on a series of experiments by our research group investigating Toll-like receptor (TLR)-mediated signaling in septic mouse models. Previous studies demonstrated organ-specific and time-dependent upregulation of TLR2, TLR3, TLR4, and TLR7 in the lung, intestine, liver, and kidney, implicating these receptors in the pathogenesis of sepsis-related acute organ injury, including ARDS, hepatic dysfunction, and acute kidney disease [7,9,10]. In addition, coordinated activation of downstream TLR signaling components, inflammatory mediators, interferons, and stem cell-related genes in the myocardium highlighted a sustained inflammatory response during sepsis [4].

Building on this body of work, the present study examines a comprehensive panel of genes involved in MYD88- and TRIF-dependent signaling pathways during the early and late phases of sepsis, using the cecal ligation and puncture (CLP) model in intestinal, renal, hepatic, and pulmonary tissues, providing further insight into the molecular mechanisms underlying sepsis-associated multiorgan dysfunction.

Histopathological validation of organ injury has previously been reported in the same CLP experimental cohort. Earlier investigations demonstrated progressive structural damage in the lungs, liver, and kidneys of septic animals, including inflammatory cell infiltration, edema, hepatocellular injury, cholestasis, tubular dilation, and tissue degeneration. These findings support the biological validity of the model and provide independent evidence supporting the molecular alterations observed in the present study. Accordingly, the current work focused on the transcriptional characterization of MYD88- and TRIF-dependent signaling pathways using tissues derived from this previously validated experimental setting [7,9,10].

Importantly, while previous investigations from our group characterized organ-specific TLR expression patterns during sepsis, the present study extends these observations by examining downstream molecular responses across four major organs. This approach enables a comprehensive assessment of the temporal activation of MYD88- and TRIF-related signaling pathways and provides novel insights into the coordinated transcriptional responses associated with sepsis-induced multiorgan dysfunction.

All experiments were conducted in young adult (12–14-week-old) male C57BL/6J mice, corresponding to pediatric or young adolescent humans [7]. While this approach minimized biological variability and enabled standardized comparisons, it limits conclusions regarding the effects of aging and sex on septic immune responses. Given that sepsis disproportionately affects elderly populations and that aging is associated with chronic inflammation and dysregulation of protective factors such as Klotho, future studies in aged animals are warranted. In addition, well-documented sex-based differences in immune and inflammatory responses underscore the need for studies incorporating both sexes.

Overall, our results demonstrate that the CLP model of sepsis induces a robust, time-dependent activation of innate immune signaling pathways, peaking at 72 h compared with controls. Upregulation of MYD88, IRAK1, IRAK4, NF-κB, CCL4, CCL20, CCR2, IFN-β, IFN-γ, TNF-α, IL-1β, IL-2, IL-4, IL-8, IL-10, IL-18, as well as Klotho and stem cell-associated genes (KLF4, HOXA5, NANOG, and HIF-1α), is associated with the development of systemic inflammation and organ-specific responses during sepsis, potentially contributing to the pathophysiology of sepsis-induced multiorgan dysfunction.

It should be noted that mRNA expression does not necessarily reflect protein levels or functional activity. The observed transcriptional changes should therefore be interpreted as indicative of pathway activation rather than definitive evidence of functional effects. Additionally, several of the analyzed genes may not be exclusively regulated by MYD88/TRIF-dependent pathways, as some can act as downstream targets of multiple signaling cascades. This suggests that their expression may reflect broader inflammatory signaling networks beyond the MYD88/TRIF axis. Future studies integrating transcriptomic datasets from publicly available repositories (e.g., GEO) would provide valuable validation and enable broader contextualization of the findings.

While the observed transcriptional changes suggest activation of MYD88- and TRIF-related pathways during sepsis, it should be noted that alternative splicing and tissue-specific transcript variants may further modulate receptor signaling and downstream inflammatory responses [11,12,13].

The progressive increase in MYD88, IRAK1, and IRAK4 mRNA expression from 24 to 72 h indicates sustained activation of MyD88-dependent TLR signaling during sepsis. Engagement of this pathway promotes IRAK phosphorylation and downstream NF-κB activation, a central transcriptional regulator that drives the expression of cytokines and chemokines critical to the septic inflammatory response [14,15]. Consistent with this mechanism, increased expression of the chemokines CCL4 and CCL20 and the CCR2 receptor suggests enhanced recruitment of monocytes, macrophages, and other immune cells to affected tissues, contributing to pathogen clearance but also predisposing mice to tissue injury when immune activation is prolonged [16,17]. Cell-specific contributions were not directly assessed in the present study; future work incorporating cellular profiling would provide additional mechanistic insight.

The marked induction of TNF-α and IL-1β confirms a dominant proinflammatory phase, whereas increased IL-2 and IL-4 expression reflects activation of adaptive immunity and T-lymphocyte differentiation [18]. In parallel, the strong upregulation of IL-10 indicates engagement of counter-regulatory anti-inflammatory mechanisms aimed at limiting excessive immune activation [18]. The simultaneous presence of pro- and anti-inflammatory mediators highlights the characteristic immune dysregulation of sepsis, in which hyperinflammation and immunosuppression dynamically coexist [15]. Increased IL-8 expression further supports enhanced neutrophil recruitment, while induction of IL-18—an important driver of IFN-γ production—amplifies immune activation [18,19,20,21]. Accordingly, elevated IFN-β and IFN-γ expression indicates activation of TRIF-dependent signaling, primarily through TLR3 and TLR4, contributing both to antimicrobial defense and modulation of the inflammatory response [18,19,20,21].

Beyond immune activation, the significant induction of Klotho together with hIF-1α, KLF4, HOXA5, and NANOG demonstrates that sepsis also triggers molecular programs related to cellular adaptation, survival, and tissue remodeling [4]. HIF-1α, a key regulator of hypoxic signaling, reflects the profound microcirculatory dysfunction and tissue hypoxia characteristic of the septic state, whereas Klotho has been described as a cytoprotective and anti-inflammatory factor [4,22]. The transcription factors KLF4, HOXA5, and NANOG are associated with cellular plasticity, regenerative responses, and maintenance of tissue integrity, suggesting an intrinsic attempt to counterbalance inflammatory damage and promote repair during sepsis progression [4,22,23]. Mechanistically, KLF4 supports endothelial homeostasis by upregulating endothelial nitric oxide synthase, limiting adhesion molecule expression, and modulating macrophage polarization, while HOXA5 contributes to vascular stability and suppression of excessive cytokine production [4]. In contrast, NANOG has been linked to maladaptive responses, including phenotypic modulation and proliferation of vascular smooth muscle cells and disruption of cell–cell junction integrity via altered VE-cadherin localization [4].

The results demonstrated that the mRNA expression of MYD88, IRAK1, IRAK4, NF-κB, CCL4, CCL20, CCR2, IFN-β, IFN-γ, TNF-α, IL-1β, IL-2, IL-4, IL-8, IL-10, IL-18, Klotho, KLF4, HOXA5, NANOG, and HIF-1α in the intestine were consistently higher compared to the kidney, liver, and lung at all examined time points (24 h, 48 h and 72 h), both in the early and late phases of CLP-induced sepsis. This pronounced upregulation in intestinal tissue likely reflects its central role as the primary site of infection and inflammatory initiation in the CLP model. Disruption of the intestinal barrier facilitates increased exposure to microbial products, leading to sustained Toll-like receptor stimulation and robust activation of both MYD88- and TRIF-dependent signaling pathways.

From all tested tissues we conclude that most genes develop a progressive increase in their expression during the successive windows of 24–48, 48–72 and 24–72 h. This suggests a continued activation of inflammatory and stress-responsive pathways during the time course of sepsis. The exceptions are the genes CCR2, TNFa, IL-2, IL-8, IL-10, IL-18 and HOXA5, where a small decrease in their elevation is observed in all tissues during the time period of 48–72 h, with a subsequent more dramatic increase in the time window of 24–72 h. These findings suggest that, despite a transient attempt to limit inflammation after 48 h, the immune response persists overall up to 72 h, reflecting the ongoing and not fully controlled nature of sepsis. An important observation is that the HIF1A gene showed an upward trend in its expression in all time windows of the intestinal and lung tissue. In the kidney and liver, we do not notice this, since a small drop in the increase is observed in the period of 48–72 h, with a subsequent more pronounced increase in the time course of 24–72 h. It seems that hIF-1α remains constantly increased in the intestine and lung due to persistent hypoxia, while there is a temporary adaption in the kidney and liver, which is, however, not sufficient to stop the large upward trend of the hypoxic response during sepsis.

Interpretation of the results further reveals a pronounced and consistent upregulation of IL-2, IL-10, IL-18, Klotho, HOXA5, and HIF-1α across all examined tissues, indicating coordinated enhancement of immune responses, activation of inflammation-regulatory mechanisms, and engagement of protective pathways against oxidative and hypoxic stress during sepsis [23,24,25]. Klotho exerts protective effects by reducing expression of adhesion molecules such as ICAM-1 and VCAM-1, suppressing proinflammatory mediators including IL-6, IL-8, TNF-α, and NF-κB, and attenuating oxidative stress through inhibition of Nox2-dependent superoxide production [4]. Concurrently, increased HOXA5 expression suggests transcriptional reprogramming of structural and differentiation-associated genes, consistent with its established roles in organogenesis and cellular identity, while strong HIF-1α activation reflects metabolic and vascular adaptation to hypoxia and cellular stress [22,26,27].

Finally, organ-specific patterns of inflammatory signaling were observed. IL-8 expression peaked at 24–48 h in intestinal, renal, and pulmonary tissues, followed by a modest decline, whereas hepatic IL-8 levels remained elevated up to 72 h. This sustained hepatic response likely reflects persistent activation of Kupffer cells, which play a central role in maintaining systemic inflammation during sepsis [28]. Kupffer cells are key mediators of early cytokine release and are critically involved in sepsis-induced liver injury through sustained production of TNF-α, IL-1, IL-6, IFN-γ, IL-8, and monocyte chemoattractant proteins [29].

Comparative analysis across the four tissues demonstrated upregulation of all examined genes in septic models; however, the magnitude of induction was consistently less pronounced in the kidney. This attenuated response may reflect the lower basal expression of TLRs in renal tissue compared with other organs [30]. In addition, the kidney possesses robust anti-inflammatory and cytoprotective mechanisms, notably Klotho, which suppresses NF-κB signaling and limits cytokine production [31,32]. The relatively reduced inflammatory response may also be explained by tissue-specific immune composition, as the kidney contains fewer resident immune cells than the intestine, liver, and lung [28]. Renal parenchyma is dominated by tubular epithelial cells, with comparatively sparse populations of macrophages and dendritic cells, resulting in a blunted early inflammatory response [33]. In contrast, the intestine harbors the gut-associated lymphoid tissue (GALT), the liver contains abundant Kupffer cells, and the lung is rich in alveolar macrophages, dendritic cells, and neutrophils, collectively enabling rapid and robust TLR pathway activation during sepsis [28,33,34].

### Limitations of the Study

Despite the clear activation of MYD88- and TRIF-dependent signaling pathways during the early and intermediate phases of sepsis, several limitations should be considered. Regarding the methodology of the study, it should be noted that as a secondary analysis, direct validation measures such as histopathological assessment or additional biomarker evaluation were not reassessed and therefore remain unconfirmed. RNA quality was evaluated based on purity and amplification consistency; although RNA integrity number (RIN) values were not formally recorded, the consistent Ct values and amplification efficiency support the adequacy of RNA for qRT-PCR analysis. In addition, the reliance on previously generated aggregated datasets limited access to individual raw data points, precluding detailed graphical replotting and individual-level data visualization.

Second, this study relied exclusively on the cecal ligation and puncture (CLP) murine model. Although CLP is regarded as the most clinically relevant preclinical model, it cannot fully capture the heterogeneity of human sepsis with respect to etiology, microbial burden, disease severity, comorbidities, and therapeutic interventions [35]. Therefore, extrapolation of these findings to diverse clinical settings should be undertaken with caution.

Third, the study was conducted exclusively in young male mice. Given that sex- and age-related differences significantly influence immune responses and sepsis outcomes, the findings may not be fully generalizable to female or aged populations.

Fourth, gene expression analyses were performed on whole tissue samples. As a result, the data do not provide information regarding cell-specific contributions to the observed transcriptional changes. Future studies incorporating single-cell or cell-sorting approaches would help clarify the cellular sources of these responses.

Fifth, the study focused on mRNA expression levels without corresponding assessment of protein expression or functional activity. As mRNA levels do not always correlate with protein abundance or biological function, the findings should be interpreted as indicative of transcriptional regulation rather than definitive evidence of pathway activation. Furthermore, gene expression was quantified at the total transcript level and did not distinguish between alternative splice variants or transcript isoforms. Since alternative splicing has been shown to influence innate immune signaling and receptor function in a tissue-specific manner, the observed transcriptional changes may reflect the combined expression of multiple transcript variants [36,37]. Future studies employing RNA sequencing or isoform-specific approaches are warranted to further characterize transcript diversity during sepsis.

Sixth, although the selected gene panel was designed to capture key components of MYD88- and TRIF-dependent signaling pathways, it does not represent the full complexity of the transcriptional landscape. More comprehensive approaches, such as RNA sequencing, would provide broader insight into sepsis-specific molecular signatures.

Finally, the selected time points (24, 48, and 72 h) primarily capture early and intermediate phases of the septic response. Later stages associated with immune exhaustion, persistent inflammation, or recovery were not evaluated, preventing full characterization of the temporal evolution of immune dysregulation. Additional time points could further refine the temporal characterization of molecular responses.

## 5. Conclusions

In conclusion, the findings of the present study support that sepsis is a multidimensional, time-evolving and tissue-dependent process, in which prolonged activation of immune and hypoxic pathways contributes to the development of multiorgan dysfunction. Mapping these molecular changes provides valuable information for understanding the pathogenesis of sepsis and highlights potential molecular targets for future therapeutic interventions and time-targeted treatment strategies.

## Figures and Tables

**Table 1:** Time-dependent changes of MYD88, IRAK1, IRAK4, NF-κB, CCL4, CCL20, CCR2, IFN-β, IFN-γ, TNF-α, IL-1β, IL-2, IL-4, IL-8, IL-10, IL-18, Klotho, KLF4, HOXA5, NANOG and HIF1α gene expressions in the intestinal tissue of septic mice across defined time intervals (24–48 h, 48–72 h, and 24–72 h). Data are presented as mean difference (MD) in relative mRNA expression between the indicated time points, along with 95% confidence level (CI). Across all genes, statistically significant negative MD values indicate a consistent time-dependent increase in gene expression with larger absolute MD values reflecting greater magnitude of change between intervals. Statistical significance was assessed using one-way ANOVA and is denoted as *** (*p* < 0.001).

Time Intervals Genes	24–48 h	48–72 h	24–72 h
*MYD88*	−4.92(−8.87 to −0.96) ***	−9.14 (−13.10 to −5.18) ***	−14.07 (−18.02 to −10.11) ***
*IRAK1*	−5.34 (−9.59 to −1.08) ***	−9.76 (−14.02 to −5.50) ***	−15.11 (−19.36 to −10.85) ***
*IRAK4*	−5.85 (−10.42 to −1.28) ***	−10.58 (−15.14 to −6.00) ***	−16.43 (−21.00 to −11.87) ***
*NFkB*	−6.73 (−11.98 to −1.49) ***	−12.16 (−17.41 to −6.91) ***	−18.90 (−24.15 to −13.66) ***
*CCL4*	−6.83 (−12.36 to −1.31) ***	−12.48 (−18.01 to −6.96) ***	−19.32 (−24.84 to −13.80) ***
*CCL20*	−1.76 (−3.23 to −0.29) ***	−4.71 (−6.19 to −3.24) ***	−6.48 (−7.95 to −5.00) ***
*CCR2*	−3.08 (−5.04 to −1.12) ***	−2.91 (−4.87 to −0.95) ***	−6.00 (−7.95 to −4.04) ***
*IFN-β*	−2.83 (−5.21 to −0.45) ***	−3.64 (−6.03 to −1.26) ***	−6.48 (−8.86 to −4.10) ***
*IFN-γ*	−3.34 (−5.20 to −1.47) ***	−5.97 (−7.84 to −4.11) ***	−9.31 (−11.18 to −7.45) ***
*TNFa*	−4.93 (−6.39 to −3.48) ***	−3.84 (−5.30 to −2.38) ***	−8.78 (−10.24 to −7.32) ***
*IL-1* *β*	−8.44 (−10.76 to −6.12) ***	−12.75 (−15.06 to −10.43) ***	−21.19 (−23.50 to −18.88) ***
*IL-2*	−18.03 (−21.60 to −14.45) ***	−14.23 (−17.81 to −10.65) ***	−32.26 (−35.84 to −28.68) ***
*IL-4*	−3.85 (−6.87 to −0.83) ***	−12.27 (−15.29 to −9.24) ***	−16.12 (−19.14 to −13.10) ***
*IL-8*	−17.36 (−19.89 to −14.82) ***	−2.77 (−0.24 to −5.31) ***	−14.58 (−17.11 to −12.05) ***
*IL-10*	−18.80 (−23.54 to −14.06) ***	−10.30 (−15.04 to −5.56) ***	−29.10 (−33.84 to −24.36) ***
*IL-18*	−17.26 (−19.33 to −15.18) ***	−15.67 (−17.74 to −13.59) ***	−32.92 (−35.00 to −30.85) ***
*KLOTHO*	−10.81 (−14.59 to −7.03) ***	−20.92 (−24.70 to −17.14) ***	−31.73 (−35.51 to −27.95) ***
*KLF4*	−5.28 (−8.65 to −1.90) ***	−14.67 (−18.05 to −11.29) ***	−19.95 (−23.33 to −16.57) ***
*HOXA5*	−19.39 (−22.77 to −16.00) ***	−16.25 (−19.64 to −12.87) ***	−35.64 (−39.03 to −32.25) ***
*NANOG*	−9.44 (−12.15 to −6.74) ***	−13.08 (−15.78 to −10.38) ***	−22.53(−25.23 to −19.83) ***
*HIF1α*	−18.54 (−25.05 to −12.03) ***	−20.99 (−27.50 to −14.48) ***	−39.53 (−46.04 to −33.02) ***

**Table 2:** Time-dependent changes of MYD88, IRAK1, IRAK4, NF-κB, CCL4, CCL20, CCR2, IFN-β, IFN-γ, TNF-α, IL-1β, IL-2, IL-4, IL-8, IL-10, IL-18, Klotho, KLF4, HOXA5, NANOG and HIF1α gene expressions in the kidney tissue of septic mice across defined time intervals (24–48 h, 48–72 h, and 24–72 h). Data are presented as mean difference (MD) in relative mRNA expression between the indicated time points, along with 95% confidence level (CI). Across all genes, statistically significant negative MD values indicate a consistent time-dependent increase in gene expression with larger absolute MD values reflecting greater magnitude of change between intervals. Statistical significance was assessed using one-way ANOVA and is denoted as *** (*p* < 0.001).

Time Intervals Genes	24–48 h	48–72 h	24–72 h
*MYD88*	−2.89 (−5.23 to −0.55) ***	−5.38 (−7.72 to −3.04) ***	−8.27 (−10.61 to −5.93) ***
*IRAK1*	−3.14 (−5.65 to −0.62) ***	−5.74 (−8.26 to −3.23) ***	−8.88 (−11.40 to −6.37) ***
*IRAK4*	−3.44 (−6.13 to −0.75) ***	−6.22 (−8.91 to −3.52) ***	−9.66 (−12.36 to −6.97) ***
*NFkB*	−3.96 (−7.05 to −0.87) ***	−7.15 (−10.25 to −4.06) ***	−11.12 (−14.21 to −8.02) ***
*CCL4*	−4.02 (−7.27 to −0.77) ***	−7.34 (−10.59 to −4.09) ***	−11.37 (−14.62 to −8.11) ***
*CCL20*	−1.03 (−2.12 to −0.04) ***	−2.77 (−3.85 to −1.69) ***	−3.81 (−4.89 to −2.73) ***
*CCR2*	−1.815 (−3.15 to −0.47) ***	−1.71 (−3.05 to −0.37) ***	−3.53 (−4.87 to −2.18) ***
*IFN-β*	−1.66 (−3.09 to −0.23) ***	−2.14 (−3.57 to −0.71) ***	−3.81 (−5.24 to −2.38) ***
*IFN-γ*	−1.96 (−3.10 to −0.82) ***	−3.51 (−4.65 to −2.37) ***	−5.48 (−6.62 to −4.34) ***
*TNFa*	−2.90 (−3.90 to −1.90) ***	−2.26 (−3.25 to −1.26) ***	−5.16 (−6.16 to −4.16) ***
*IL-1* *β*	−4.96 (−6.46 to −3.46) ***	−7.49 (−8.99 to −5.99) ***	−12.47 (−13.97 to −10.96) ***
*IL-2*	−10.60 (−12.76 to −8.44)***	−8.3 (−10.53 to −6.21) ***	−18.98 (−21.13 to −16.82) ***
*IL-4*	−2.26 (−4.12 to −0.40) ***	−7.21 (−9.07 to −5.35) ***	−9.48 (−11.34 to −7.62) ***
*IL-8*	−10.21 (−11.76 to −8.66) ***	−1.63 (−0.08 to −3.18) ***	−8.57 (−10.12 to −7.02) ***
*IL-10*	−11.06 (−13.86 to −8.25) ***	−6.05 (−8.86 to −3.25) ***	−17.12 (−19.92 to −14.31) ***
*IL-18*	−10.15 (−11.50 to −8.80) ***	−9.21 (−10.56 to −7.86) ***	−19.37 (−20.71 to −18.02) ***
*KLOTHO*	−6.36 (−8.62 to −4.10) ***	−12.31 (−14.57 to −10.05) ***	−18.67 (−20.93 to −16.41) ***
*KLF4*	−3.10 (−5.10 to −1.10) ***	−8.62 (−10.63 to −6.63) ***	−11.74 (−13.73 to −9.73) ***
*HOXA5*	−11.41 (−13.41 to −9.40) ***	−9.56 (−11.56 to −7.55) ***	−20.90 (−22.97 to −18.96) ***
*NANOG*	−6.49 (−8.63 to −4.34) ***	−6.96 (−9.11 to −4.82) ***	−13.46 (−15.60 to −11.31) ***
*HIF1α*	−13.01 (−16.75 to −9.25) ***	−12.35 (−16.09 to −8.60) ***	−25.35 (−29.10 to −21.61) ***

**Table 3:** Time-dependent changes in MYD88, IRAK1, IRAK4, NF-κB, CCL4, CCL20, CCR2, IFN-β, IFN-γ, TNF-α, IL-1β, IL-2, IL-4, IL-8, IL-10, IL-18, Klotho, KLF4, HOXA5, NANOG and HIF1α gene expressions in the liver tissue of septic mice across defined time intervals (24–48 h, 48–72 h, and 24–72 h). Data are presented as mean difference (MD) in relative mRNA expression between the indicated time points, along with 95% confidence level (CI). Across all genes, statistically significant negative MD values indicate a consistent time-dependent increase in gene expression with larger absolute MD values reflecting greater magnitude of change between intervals. Statistical significance was assessed using one-way ANOVA and is denoted as *** (*p* < 0.001).

Time Intervals Genes	24–48 h	48–72 h	24–72 h
*MYD88*	−3.76 (−6.80 to −0.72) ***	−6.99 (−10.04 to −3.95) ***	−10.76 (−13.80 to −7.716) ***
*IRAK1*	−4.08 (−7.35 to −0.81) ***	−7.46 (−10.74 to −4.19) ***	−11.55 (−14.82 to −8.283) ***
*IRAK4*	−4.47 (−7.98 to −0.97) ***	−8.08 (−11.59 to −4.58) ***	−12.57 (−16.07 to −9.06) ***
*NFkB*	−5.15 (−9.17 to −1.13) ***	−9.30 (−13.32 to −5.28) ***	−14.45 (−18.47 to −10.43) ***
*CCL4*	−5.22 (−9.45 to −1.00) ***	−9.54 (−13.77 to −5.32) ***	−14.77 (−19.00 to −10.55) ***
*CCL20*	−1.75 (−3.22 to −0.28) ***	−4.69 (−6.15 to −3.22) ***	−6.44 (−7.91 to −4.97) ***
*CCR2*	−3.06 (−5.02 to −1.11) ***	−2.89 (−4.85 to −0.94) ***	−5.96 (−7.91 to −4.01) ***
*IFN-β*	−2.16 (−3.98 to −0.34) ***	−2.79 (−4.61 to −0.97) ***	−4.95 (−6.77 to −3.13) ***
*IFN-γ*	−2.55 (−3.98 to −1.12) ***	−4.57 (−6.00 to −3.14) ***	−7.12 (−8.55 to −5.69) ***
*TNFa*	−3.77 (−4.90 to −2.65) ***	−2.93 (−4.06 to −1.81) ***	−6.71 (−7.84 to −5.58) ***
*IL-1* *β*	−6.45 (−8.23 to −4.67) ***	−9.74 (−11.53 to −7.96) ***	−16.20 (−17.98 to −14.43) ***
*IL-2*	−13.78 (−16.53 to −11.04) ***	−10.88 (−13.63 to −8.14) ***	−24.67 (−27.41 to −21.93) ***
*IL-4*	−2.94 (−4.26 to −0.63) ***	−9.38 (−11.70 to −7.06) ***	−12.33 (−14.64 to −10.01) ***
*IL-8*	−11.15 (−13.09 to −9.20) ***	−2.12 (−4.06 to −0.17) ***	−13.27 (−15.22 to −11.33) ***
*IL-10*	−14.38 (−18.00 to −10.75) ***	−7.87 (−11.50 to −4.25) ***	−22.25 (−25.88 to −18.63)***
*IL-18*	−13.20 (−14.79 to −11.60) ***	−11.98 (−13.58 to −10.38) ***	−25.18 (−26.78 to −23.58) ***
*KLOTHO*	−8.26 (−11.16 to −5.37) ***	−16.00 (−18.89 to −13.11) ***	−24.27 (−27.16 to −21.37) ***
*KLF4*	−4.03 (−6.63 to −1.44) ***	−11.22 (−13.82 to −8.62) ***	−15.26 (−17.85 to −12.66) ***
*HOXA5*	−14.83 (−17.42 to −12.23) ***	−12.43 (−15.03 to −9.83) ***	−27.25 (−29.85 to −24.66) ***
*NANOG*	−8.43 (−11.24 to −5.64) ***	−9.05 (−11.85 to −6.25) ***	−17.49 (−20.29 to −14.70) ***
*HIF1α*	−16.91 (−21.78 to −12.03) ***	−16.05 (−20.92 to −11.18) ***	−32.96 (−37.83 to −28.08) ***

**Table 4:** Time-dependent changes in MYD88, IRAK1, IRAK4, NF-κB, CCL4, CCL20, CCR2, IFN-β, IFN-γ, TNF-α, IL-1β, IL-2, IL-4, IL-8, IL-10, IL-18, Klotho, KLF4, HOXA5, NANOG and HIF1α gene expressions in the lung tissue of septic mice across defined time intervals (24–48 h, 48–72 h, and 24–72 h). Data are presented as mean difference (MD) in relative mRNA expression between the indicated time points, along with 95% confidence level (CI). Across all genes, statistically significant negative MD values indicate a consistent time-dependent increase in gene expression with larger absolute MD values reflecting greater magnitude of change between intervals. Statistical significance was assessed using one-way ANOVA and is denoted as *** (*p* < 0.001) indicating highly significant differences between compared time points.

Time Intervals Genes	24–48 h	48–72 h	24–72 h
*MYD88*	−4.42 (−7.99 to −0.86) ***	−8.23 (−11.79 to −4.67) ***	−12.66 (−16.22 to −9.09) ***
*IRAK1*	−4.80 (−8.64 to −0.97) ***	−8.79 (−12.62 to −4.95) ***	−13.60 (−17.43 to −9.76) ***
*IRAK4*	−5.27 (−9.37 to −1.16) ***	−9.51 (−13.63 to −5.41) ***	−14.79 (−18.90 to −10.68) ***
*NFkB*	−6.06 (−10.78 to −1.34) ***	−10.95 (−15.67 to −6.22) ***	−17.01 (−21.73 to −12.29) ***
*CCL4*	−6.15 (−11.12 to −1.18) ***	−11.24 (−16.20 to −6.26) ***	−17.39 (−22.36 to −12.42) ***
*CCL20*	−1.58 (−2.91 to −0.26) ***	−4.24 (−5.57 to −2.91) ***	−5.83 (−7.16 to −4.50) ***
*CCR2*	−2.77 (−4.53 to −1.01) ***	−2.62 (−4.38 to −0.86) ***	−5.40 (−7.16 to −3.63) ***
*IFN-β*	−2.55 (−4.69 to −0.40) ***	−3.28 (−5.42 to −1.14) ***	−5.83 (−7.97 to −3.69) ***
*IFN-γ*	−3.00 (−4.68 to −1.33) ***	−5.38 (−7.05 to −3.70) ***	−8.38 (−10.06 to −6.71) ***
*TNFa*	−4.44 (−5.75 to −3.13) ***	−3.45 (−4.76 to −2.14) ***	−7.90 (−9.21 to −6.59) ***
*IL-1* *β*	−7.59 (−9.68 to −5.51) ***	−11.47 (−13.56 to −9.38) ***	19.07 (−21.15 to −16.99) ***
*IL-2*	−16.22 (−19.44 to −13.00) ***	−12.81 (−16.03 to −9.58) ***	−29.03 (−32.25 to −25.81) ***
*IL-4*	−3.46 (−6.18 to −0.74) ***	−11.04 (−13.76 to −8.32) ***	−14.51 (−17.23 to −11.79) ***
*IL-8*	−17.36 (−19.89 to −14.82) ***	2.77 (0.24 to 5.31) ***	−14.58 (−17.11 to −12.05) ***
*IL-10*	−16.92 (−21.19 to −12.65) ***	−9.26 (−13.54 to −5.00) ***	−26.19 (−30.45 to −21.92) ***
*IL-18*	−15.53 (−17.40 to −13.66) ***	−14.10 (−15.97 to −12.23) ***	−29.63 (−31.50 to −27.76) ***
*KLOTHO*	−9.73 (−13.13 to −6.33) ***	−18.83 (−22.23 to −15.43) ***	−28.56 (−31.96 to −25.16) ***
*KLF4*	−4.75 (−7.79 to −1.71) ***	−13.20 (−16.24 to −10.17) ***	−17.96 (−20.99 to −14.92) ***
*HOXA5*	−17.45 (−20.49 to −14.41) ***	−14.63 (−17.67 to −11.58) ***	−32.08 (−35.12 to −29.03) ***
*NANOG*	−8.50 (−10.93 to −6.07) ***	−11.77 (−14.21 to −9.34) ***	−20.28 (−22.71 to −17.85) ***
*HIF1α*	−16.69 (−22.54 to −10.83) ***	−18.89 (−24.75 to −13.03) ***	−35.58 (−41.43 to −29.72) ***

## Data Availability

The datasets generated and/or analyzed during the current study are not publicly available at this stage, as they are part of ongoing research, but are available from the corresponding author on reasonable request.

## Supplementary Materials

The following supporting information can be downloaded at: https://www.mdpi.com/article/10.3390/cimb48070660/s1.
