# Supplementary material for: MYD88/TRIF Signaling, Pluripotency and Klotho Regulation in the Intestine, Kidneys, Liver, and Lungs of a Septic Mouse Model
Source: Curr Issues Mol Biol. 2026 Jun 26;48(7):660. doi: 10.3390/cimb48070660 (PMC13407125; doi:10.3390/cimb48070660)
Supplement: Supplementary file 1 [file cimb-48-00660-s001.zip › cimb-4357464-supplementary.pdf]

Supplementary Table S1. Primers used for QRT PCR

Primer<br><br>Gene	Forward	Reverse	NCBI reference sequence
MYD88	ACCTGTGTCTGGTCCATTGCCA	GCTGAGTGCAAACTTGGTCTGG	NM_010851.3
IRAK1	GGACTTCCACAGTTCGAGGTAC	GGTCTTTGCACCTTGTGTCCTC	NM_008363
IRAK4	GGTCCAGATTGAGCTGTTTGCC	GTTTGTGCCACTGTTGCCGCTT	NM_029926
NF-kB	TCCTGTTCGAGTCTCCATGCAG	GGTCTCATAGGTCCTTTTGCGC	NM_009045
CCL4	ACCCTCCCACTTCCTGCTGTTT	CTGTCTGCCTCTTTTGGTCAGG	NM_013652
CCL20	GTGGGTTTCACAAGACAGATGGC	CCAGTTCTGCTTTGGATCAGCG	NM_016960
CCR2	GCTGTGTTTGCCTCTCTACCAG	CAAGTAGAGGCAGGATCAGGCT	NM_009915
IFN-β	GCCTTTGCCATCCAAGAGATGC	ACACTGTCTGCTGGTGGAGTTC	NM_010510
IFN-γ	CAGCAACAGCAAGGCGAAAAAGG	TTTCCGCTTCCTGAGGCTGGAT	NM_008337
TNF-α	GGTGCCTATGTCTCAGCCTCTT	GCCATAGAACTGATGAGAGGGAG	NM_013693
IL-1b	TGGACCTTCCAGGATGAGGACA	GTTCATCTCGGAGCCTGTAGTG	NM_008361
IL-2	GCGGCATGTTCTGGATTTGACTC	CCACCACAGTTGCTGACTCATC	NM_008366
IL-4	ATCATCGGCATTTTGAACGAGGTC	ACCTTGGAAGCCCTACAGACGA	NM_021283
IL-8	GGTGATATTCGAGACCATTTACTG	GCCAACAGTAGCCTTCACCCAT	NM_011339
IL-10	CGGGAAGACAATAACTGCACCC	CGGTTAGCAGTATGTTGTCCAGC	NM_010548
IL-18	GACAGCCTGTGTTCGAGGATATG	TGTTCTTACAGGAGAGGGTAGAC	NM_008360
A- Klotho	CCTCCTTTACCTGAAAACCAGCC	CCACAGATAGACATTCGGGTCAG	NM_013823.1
KLF4	CTATGCAGGCTGTGGCAAAACC	TTGCGGTAGTGCCTGGTCAGTT	NM_010637
HOXA5	CGCAAGCTGCACATTAGTCACG	GAGAGGCAAAGGGCATGAGCTA	NM_010453
NANOG	GAACGCCTCATCAATGCCTGCA	GAATCAGGGCTGCCTTGAAGAG	NM_028016
HIF1α	CCTGCACTGAATCAAGAGGTTGC	CCATCAGAAGGACTTGCTGGCT	NM_010431
GAPDH	CATCACTGCCACCCAGAAGACTG	ATGCCAGTGAGCTTCCCGTTCAG	NM_008084

Supplementary Table S2. Intestinal tissue

Time Points<br>Groups	24h	48h	72h
**MYD88**			
Control	0.68±0.19	0.64±0.15	0.55±0.15
Septic	4.85±2.09[#]	9.77±3.08[#,a]	18.92±4.05[#,a,b,c]
**IRAK1**			
Control	0.57±0.19	0.54±0.21	0.35±0.12
Septic	5.28±2.27[#]	10.62±3.28[#,a]	20.39±4.38[#,a,b,c]
**IRAK4**			
Control	0.59±0.26	0.52±0.28	0.65±0.24
Septic	5.67±2.45[#]	11.53±3.64[#,a]	22.11±4.58[#,a,b,c]
**NFKB**			
Control	0.71±0.23	0.62±0.23	0.75±0.21
Septic	6.52±2.82[#]	13.27±4.19[#,a]	25.43±5.27[#,a,b,c]
**CCL4**			
Control	0.69±0.20	0.59±0.11	0.55±0.10
Septic	7.01±2.34[#]	13.86±2.61[#,a]	26.34±6.85[#,a,b,c]
**CCL20**			
Control	0.73±0.15	0.76±0.14	0.74±0.11
Septic	6.43±0.77[#]	8.19±1.03[#,a]	12.91±1.58[#,a,b,c]
**CCR2**			
Control	0.75±0.12	0.66±0.04	0.64±0.13
Septic	7.82±1.46[#]	10.91±1.72[#,a]	13.82±1.51[#,a,b,c]
**IFNB**			
Control	0.83±0.10	0.80±0.10	0.74±0.12
Septic	7.59±1.17[#]	10.43±1.76[#,a]	14.08±2.55[#,a,b,c]
**IFNG**			
Control	0.83±0.10	0.62±0.13	0.71±0.12
Septic	5.38±1.73[#]	8.72±1.21[#,a]	14.71±1.48[#,a,b,c]
**TNFA**			
Control	0.80±0.15	0.70±0.08	0.71±0.10
Septic	5.47±1.01[#]	10.41±1.13[#,a]	14.25±1.33[#,a,b,c]
**IL1B**			
Control	0.81±0.09	0.77±0.13	0.76±0.09
Septic	6.85±1.31[#]	15.30±1.64[#,a]	28.05±2.44[#,a,b,c]
**IL2**			
Control	0.77±0.15	0.71±0.10	0.75±0.10
Septic	6.86±1.44[#]	24.89±4.50[#,a]	39.12±1.57[#,a,b,c]
**IL4**			
Control	0.87±0.08	0.71±0.18	0.72±0.15
Septic	10.03±1.38[#]	13.88±1.44[#,a]	26.15±3.70[#,a,b,c]
**IL8**			
Control	0.77±0.12	0.81±0.06	0.69±0.07
Septic	8.86±1.60[#]	26.23±2.59[#,a]	23.45±1.78[#,a,b,c]
**IL10**			
Control	0.76±0.11	0.83±0.04	0.74±0.12
Septic	8.95±2.09[#]	27.75±1.80[#,a]	38.05±6.00[#,a,b,c]
**IL18**			
Control	0.79±0.10	0.76±0.07	0.67±0.06
Septic	6.20±1.26[#]	23.46±2.32[#,a]	39.13±1.16[#,a,b,c]

**KLOTHO**			
Control	0.73±0.17	0.81±0.09	0.73±0.06
Septic	8.24±1.34[#]	19.06±4.15[#,a]	39.98±2.94[#,a,b,c]
**KLF4**			
Control	0.53±0.14	0.70±0.17	0.73±0.19
Septic	5.62±1.01[#]	10.90±1.30[#,a]	25.57±4.40[#,a,b,c]
**HOXA5**			
Control	0.54±0.15	0.70±0.17	0.73±0.19
Septic	6.29±1.69[#]	25.68±3.62[#,a]	41.94±2.48[#,a,b,c]
**NANOG**			
Control	0.54±0.13	0.33±0.05	0.46±0.07
Septic	3.65±1.06[#]	13.10±1.04[#,a]	26.18±3.45[#,a,b,c]
**HIF1A**			
Control	0.65±0.16	0.60±0.13	0.70±0.12
Septic	8.44±2.03[#]	26.98±3.77[#,a]	47.97±7.99[#,a,b,c]

Time-dependent mRNA expression of MYD88- and TRIF-related signaling molecules, cytokines, chemokines, and stem cell–associated genes in intestine tissue of control and septic mice. Values are expressed as mean ± SD, N=12 for each experimental group. Statistical significance (p<0.05) between groups at the same time point is indicated as follows: (#) Sham vs S [24,48,72h], (a) S24 vs S48, (b) S24 vs S72, (c) S48 s S72.

Supplementary Table S3. Kidney tissue

**Time Points Groups**	**24h**	**48h**	**72h**
**MYD88**			
Control	0.50±0.22	0.50±0.27	0.30±0.11
Septic	2.85±1.23[#]	5.75±1.81[#,a]	11.13±2.38[#,a,b,c]
**IRAK1**			
Control	0.48±0.21	0.52±0.28	0.31±0.11
Septic	3.10±1.33[#]	6.24±1.93[#,a]	11.99±2.57[#,a,b,c]
**IRAK4**			
Control	0.49±0.21	0.50±0.27	0.32±0.11
Septic	3.34±1.44[#]	6.78±2.14[#,a]	13.01±2.69[#,a,b,c]
**NFKB**			
Control	0.49±0.21	0.50±0.27	0.32±0.11
Septic	3.84±1.66[#]	7.80±2.46[#,a]	14.96±3.10[#,a,b,c]
**CCL4**			
Control	0.43±0.13	0.39±0.18	0.29±0.03
Septic	4.12±1.37[#]	8.15±1.53[#,a]	15.49±4.02[#,a,b,c]
**CCL20**			
Control	1.58±0.25	1.61±0.66	1.97±0.58
Septic	3.78±0.45[#]	4.82±0.60[#,a]	7.59±0.93[#,a,b,c]
**CCR2**			
Control	2.31±0.59	2.07±0.49	1.49±0.58
Septic	4.60±0.86[#]	6.41±1.01[#,a]	8.13±0.89[#,a,b,c]

**IFNβ**			
Control	1.58±0.27	1.67±0.21	1.55±0.19
Septic	4.46±0.69[#]	6.13±1.03[#,a]	8.28±1.50[#,a,b,c]
**IFNγ**			
Control	1.70±0.27	1.46±0.30	1.60±0.20
Septic	3.16±1.01[#]	5.13±0.71[#,a]	8.65±0.87[#,a,b,c]
**TNFA**			
Control	1.37±0.29	2.03±0.59	1.75±0.28
Septic	3.22±0.59[#]	6.12±0.66[#,a]	8.38±0.78[#,a,b,c]
**IL1B**			
Control	1.73±0.27	2.15±0.82	1.58±0.17
Septic	4.03±0.77[#]	9.00±0.96[#,a]	16.50±1.43[#,a,b,c]
**IL2**			
Control	1.52±0.34	1.38±0.24	1.93±0.49
Septic	4.03±0.85[#]	14.64±2.65[#,a]	23.01±0.92[#,a,b,c]
**IL4**			
Control	2.14±0.28	1.91±0.67	1.69±0.25
Septic	5.90±0.81[#]	8.16±0.84[#,a]	15.38±2.17[#,a,b,c]
**IL8**			
Control	1.41±0.24	1.66±0.20	1.93±0.49
Septic	5.21±0.94[#]	15.43±1.52[#,a]	13.79±1.04[#,a,b,c]
**IL10**			
Control	1.90±0.34	1.73±0.10	1.69±0.25
Septic	5.26±1.23[#]	16.33±1.06[#,a]	22.38±3.53[#,a,b,c]
**IL18**			
Control	1.46±0.18	1.87±0.72	1.47±0.29
Septic	3.65±0.74[#]	13.80±1.36[#,a]	23.02±0.68[#,a,b,c]
**KLOTHO**			
Control	1.66±0.48	1.75±0.20	1.57±0.24
Septic	4.85±0.78[#]	11.21±2.44[#,a]	23.52±1.73[#,a,b,c]
**KLF4**			
Control	0.21±0.07	0.34±0.16	0.47±0.30
Septic	3.30±0.59[#]	6.41±0.76[#,a]	15.04±2.58[#,a,b,c]
**HOXA5**			
Control	0.23±0.07	0.34±0.16	0.47±0.30
Septic	3.70±0.99[#]	15.11±2.13[#,a]	24.67±1.46[#,a,b,c]
**NANOG**			
Control	0.19±0.07	0.16±0.03	0.16±0.03
Septic	1.21±0.09[#]	7.70±0.61[#,a]	14.67±2.92[#,a,b,c]
**HIF1A**			
Control	0.19±0.05	0.18±0.04	0.21±0.04
Septic	2.86±0.47[#]	15.87±2.22[#,a]	28.22±4.70[#,a,b,c]

Time-dependent mRNA expression of MYD88- and TRIF-related signaling molecules, cytokines, chemokines, and stem cell–associated genes in kidney tissue of control and septic mice. Values are expressed as mean ± SD, N=12 for each experimental group. Statistical significance ($p<0.05$) between groups at the same time point is indicated as follows: (#) Sham vs S [24,48,72h], (a) S24 vs S48, (b) S24 vs S72, (c) S48 s S72.

Supplementary Table S4. Liver tissue

Time Points<br>Groups	24h	48h	72h
**MYD88**			
Control	0.65±0.28	0.66±0.35	0.40±0.15
Septic	3.71±1.60[#]	7.47±2.36[#,a]	14.47±3.10[#,a,b,c]
**IRAK1**			
Control	0.63±0.27	0.67±0.36	0.41±0.15
Septic	4.03±1.74[#]	8.12±2.51[#,a]	15.59±3.35[#,a,b,c]
**IRAK4**			
Control	0.63±0.28	0.65±0.35	0.41±0.15
Septic	4.34±1.88[#]	8.82±2.79[#,a]	16.91±3.50[#,a,b,c]
**NFKB**			
Control	0.63±0.28	0.65±0.35	0.41±0.15
Septic	4.99±2.16[#]	10.14±3.20[#,a]	19.45±4.03[#,a,b,c]
**CCL4**			
Control	0.56±0.17	0.50±0.24	0.38±0.04
Septic	5.36±1.79[#]	10.60±1.99[#,a]	20.14±5.23[#,a,b,c]
**CCL20**			
Control	0.63±0.10	0.68±0.22	0.87±0.14
Septic	6.39±0.76[#]	8.14±1.02[#,a]	12.84±1.57[#,a,b,c]
**CCR2**			
Control	0.63±0.20	0.60±0.13	0.59±0.17
Septic	7.77±1.45[#]	10.84±1.71[#,a]	13.74±1.50[#,a,b,c]
**IFNB**			
Control	0.86±0.06	0.78±0.08	0.71±0.04
Septic	5.80±0.89[#]	7.97±1.34[#,a]	10.76±1.95[#,a,b,c]
**IFNG**			
Control	0.76±0.12	0.75±0.15	0.80±0.10
Septic	4.11±1.32[#]	6.67±0.92[#,a]	11.25±1.13[#,a,b,c]
**TNFA**			
Control	0.76±0.16	0.62±0.18	0.72±0.11
Septic	4.18±0.77[#]	7.96±0.87[#,a]	10.90±1.01[#,a,b,c]
**IL1B**			
Control	0.78±0.12	0.65±0.25	0.83±0.09
Septic	5.24±1.00[#]	11.70±1.25[#,a]	21.45±1.86[#,a,b,c]
**IL2**			
Control	0.76±0.17	0.82±0.14	0.70±0.18
Septic	5.25±1.10[#]	19.03±3.44[#,a]	29.92±1.20[#,a,b,c]
**IL4**			
Control	0.85±0.11	0.62±0.22	0.82±0.12
Septic	7.67±1.05[#]	10.62±1.10[#,a]	20.00±2.83[#,a,b,c]
**IL8**			
Control	0.74±0.12	0.83±0.10	0.70±0.18
Septic	6.78±1.22[#]	17.93±1.36[#,a]	20.05±1.98[#,a,b,c]
**IL10**			
Control	0.75±0.14	0.76±0.04	0.80±0.12
Septic	6.84±1.60[#]	21.22±1.37[#,a]	29.10±4.58[#,a,b,c]

**IL18**			
Control	0.81±0.10	0.61±0.23	0.71±0.14
Septic	4.74±0.96#	17.94±1.77#,a	29.92±0.89#,a,b,c
**KLOTHO**			
Control	0.64±0.18	0.85±0.10	0.76±0.11
Septic	6.30±1.02#	14.57±3.17#,a	30.57±2.25#,a,b,c
**KLF4**			
Control	0.62±0.23	0.50±0.23	0.47±0.30
Septic	4.29±0.77#	8.33±0.99#,a	19.56±3.36#,a,b,c
**HOXA5**			
Control	0.57±0.17	0.50±0.23	0.66±0.22
Septic	4.81±1.29#	19.64±2.77#,a	32.07±1.90#,a,b,c
**NANOG**			
Control	0.56±0.20	0.74±0.16	0.74±0.16
Septic	1.57±0.12#	10.02±0.79#,a	19.07±3.80#,a,b,c
**HIF1A**			
Control	0.66±0.18	0.70±0.15	0.79±0.15
Septic	3.72±0.62#	20.64±2.88#,a	36.69±6.11#,a,b,c

Time-dependent mRNA expression of MYD88- and TRIF-related signaling molecules, cytokines, chemokines, and stem cell–associated genes in liver tissue of control and septic mice. Values are expressed as mean ± SD, N=12 for each experimental group. Statistical significance ($p<0.05$) between groups at the same time point is indicated as follows: (#) Sham vs S [24,48,72h], (a) S24 vs S48, (b) S24 vs S72, (c) S48 s S72.

Supplementary Table S5. Lung tissue

Time Points Groups	24h	48h	72h
**MYD88**			
Control	0.76±0.19	0.71±0.10	0.64±0.12
Septic	4.36±1.88#	8.79±2.77#,a	17.03±3.65#,a,b,c
**IRAK1**			
Control	0.72±0.21	0.66±0.17	0.58±0.17
Septic	4.75±2.04#	9.56±2.95#,a	18.35±3.94#,a,b,c
**IRAK4**			
Control	0.74±0.23	0.70±0.18	0.81±0.17
Septic	5.11±2.21#	10.38±3.28#,a	19.90±4.12#,a,b,c
**NFKB**			
Control	0.77±0.20	0.81±0.17	0.85±0.13
Septic	5.87±2.54#	11.94±3.77#,a	22.89±4.74#,a,b,c
**CCL4**			
Control	0.81±0.17	0.75±0.12	0.74±0.07
Septic	6.31±2.10#	12.47±2.35#,a	23.70±6.16#,a,b,c
**CCL20**			
Control	0.69±0.18	0.76±0.11	0.75±0.10
Septic	5.78±0.69#	7.37±0.92#,a	11.62±1.42#,a,b,c

**CCR2**			
Control	0.64±0.11	0.65±0.12	0.74±0.06
Septic	7.03±1.32$^{\#}$	9.81±1.55$^{\#,a}$	12.44±1.36$^{\#,a,b,c}$
**IFNB**			
Control	0.69±0.10	0.61±0.11	0.81±0.08
Septic	6.83±1.05$^{\#}$	9.38±1.58$^{\#,a}$	12.67±2.29$^{\#,a,b,c}$
**IFNG**			
Control	0.75±0.13	0.64±0.06	0.80±0.06
Septic	4.84±1.56$^{\#}$	7.85±1.09$^{\#,a}$	13.23±1.34$^{\#,a,b,c}$
**TNFA**			
Control	0.74±0.13	0.72±0.07	0.79±0.08
Septic	4.92±0.91$^{\#}$	9.37±1.02$^{\#,a}$	12.83±1.19$^{\#,a,b,c}$
**IL1B**			
Control	0.78±0.13	0.73±0.08	0.83±0.08
Septic	6.17±1.18$^{\#}$	13.77±1.47$^{\#,a}$	25.24±2.19$^{\#,a,b,c}$
**IL2**			
Control	0.71±0.10	0.79±0.08	0.86±0.07
Septic	6.17±1.30$^{\#}$	22.40±4.05$^{\#,a}$	35.21±1.14$^{\#,a,b,c}$
**IL4**			
Control	0.74±0.13	0.72±0.08	0.79±0.07
Septic	9.02±1.24$^{\#}$	12.50±1.29$^{\#,a}$	23.54±3.33$^{\#,a,b,c}$
**IL8**			
Control	0.77±0.12	0.81±0.06	0.69±0.07
Septic	8.86±1.60$^{\#}$	26.23±2.59$^{\#,a}$	23.45±1.78$^{\#,a,b,c}$
**IL10**			
Control	0.76±0.12	0.76±0.09	0.82±0.09
Septic	8.05±1.88$^{\#}$	24.98±1.62$^{\#,a}$	34.25±5.40$^{\#,a,b,c}$
**IL18**			
Control	0.74±0.11	0.75±0.08	0.81±0.07
Septic	5.58±1.13$^{\#}$	21.11±2.09$^{\#,a}$	35.22±1.04$^{\#,a,b,c}$
**KLOTHO**			
Control	0.75±0.12	0.70±0.09	0.80±0.07
Septic	7.42±1.20$^{\#}$	17.15±3.74$^{\#,a}$	35.98±2.64$^{\#,a,b,c}$
**KLF4**			
Control	0.75±0.13	0.70±0.10	0.77±0.07
Septic	5.05±0.91$^{\#}$	9.81±1.176$^{\#,a}$	23.01±3.96$^{\#,a,b,c}$
**HOXA5**			
Control	0.76±0.12	0.72±0.08	0.78±0.07
Septic	5.66±1.52$^{\#}$	23.12±3.26$^{\#,a}$	37.74±2.23$^{\#,a,b,c}$
**NANOG**			
Control	0.78±0.10	0.69±0.06	0.80±0.07
Septic	3.28±0.95$^{\#}$	11.79±0.93$^{\#,a}$	23.56±3.11$^{\#,a,b,c}$
**HIF1A**			
Control	0.76±0.12	0.71±0.07	0.84±0.08
Septic	7.59±1.83$^{\#}$	24.29±3.39$^{\#,a}$	43.18±7.19$^{\#,a,b,c}$

Time-dependent mRNA expression of MYD88- and TRIF-related signaling molecules, cytokines, chemokines, and stem cell–associated genes in lung tissue of control and septic mice. Values are expressed as mean ± SD, N=12 for each experimental group.

Statistical significance ($p<0.05$) between groups at the same time point is indicated as follows: (#) Sham vs S [24,48,72h], (a) S24 vs S48, (b) S24 vs S72, (c) S48 s S72.
